# Influenza A virus recovery, diversity, and intercontinental exchange: A multi-year assessment of wild bird sampling at Izembek National Wildlife Refuge, Alaska

**DOI:** 10.1371/journal.pone.0195327

**Published:** 2018-04-05

**Authors:** Andrew B. Reeves, Jeffrey S. Hall, Rebecca L. Poulson, Tyrone Donnelly, David E. Stallknecht, Andrew M. Ramey

**Affiliations:** 1 United States Geological Survey Alaska Science Center, Anchorage, Alaska, United States of America; 2 United States Geological Survey National Wildlife Health Center, Madison, Wisconsin, United States of America; 3 Southeastern Cooperative Wildlife Disease Study, Department of Population Health, College of Veterinary Medicine, University of Georgia, Athens, Georgia, United States of America; Oklahoma State University, UNITED STATES

## Abstract

Western Alaska is a potential point-of-entry for foreign-origin influenza A viruses (IAVs) into North America via migratory birds. We sampled waterfowl and gulls for IAVs at Izembek National Wildlife Refuge (NWR) in western Alaska, USA, during late summer and autumn months of 2011–2015, to evaluate the abundance and diversity of viruses at this site. We collected 4842 samples across five years from 25 species of wild birds resulting in the recovery, isolation, and sequencing of 172 IAVs. With the intent of optimizing sampling efficiencies, we used information derived from this multi-year effort to: 1) evaluate from which species we consistently recover viruses, 2) describe viral subtypes of isolates by host species and year, 3) characterize viral gene segment sequence diversity with respect to host species, and assess potential differences in the viral lineages among the host groups, and 4) examine how evidence of intercontinental exchange of IAVs relates to host species. We consistently recovered viruses from dabbling ducks (*Anas spp*.), emperor geese (*Chen canagica*) and glaucous-winged gulls (*Larus glaucescens*). There was little evidence for differences in viral subtypes and diversity from different waterfowl hosts, however subtypes and viral diversity varied between waterfowl host groups and glaucous-winged gulls. Furthermore, higher proportions of viral sequences from northern pintails (*Anas acuta*), emperor geese and glaucous-winged gulls were grouped in phylogenetic clades that included IAV sequences originating from wild birds sampled in Asia as compared to non-pintail dabbling ducks, a difference that may be related to intercontinental migratory tendencies of host species. Our summary of research and surveillance efforts at Izembek NWR will assist in future prioritization of which hosts to sample and swab types to collect in Alaska and elsewhere in order to maximize isolate recovery, subtype and sequence diversity for resultant viruses, and detection of evidence for intercontinental viral exchange.

## Introduction

Research and surveillance for influenza A viruses (IAVs) in wild birds has been conducted in North America for nearly 50 years [1]. However, the number and magnitude of sampling efforts increased in the United States of America (USA) following mortality events in wild birds inhabiting East Asia attributed to Goose Guandong (Gs/Gd) lineage H5N1 Highly Pathogenic (HP) IAVs beginning in 2002. Given concerns over the potential introduction of Gs/Gd lineage IAVs into North America by wild birds via migratory movements, the U.S. Geological Survey Alaska Science Center (USGS ASC) began sampling wild birds in Alaska for IAVs in 2006 as part of an interagency state-wide program [2]. While sampling efforts in Alaska have not resulted in the detection of Gs/Gd lineage IAVs, genomic characterization of low pathogenic viruses has provided important insights on viral dispersal and evolution that have been used to improve IAV sampling strategies in Alaska. For example, the consistent detection of interhemispherically reassorted IAVs in Alaska, and in particular western Alaska, provides evidence that this region likely represents an important point-of-entry for viruses from East Asia into North America [3–5].

Since 2011, the USGS ASC has focused IAV sampling on autumn-staging waterfowl and gulls within and around Izembek National Wildlife Refuge (NWR) in Southwestern Alaska to better understand the intercontinental dispersal of IAVs by migratory birds, the diversity of viruses infecting wild birds at this high latitude location, and the evolution of viruses at the confluence of divergent gene pools. Izembek NWR was selected as a research and surveillance site based upon: (1) genetic characterization of viruses resulting from sampling efforts conducted during 2006–2008 that provided support for increased detection of evidence for interhemispheric viral exchange at this site as compared to other locations in Alaska and North America [4], (2) the relatively large number of autumn staging waterfowl at this site with intercontinental migratory tendencies such as northern pintail ducks (*Anas acuta*) and emperor geese (*Chen canagica*) [6–8], and (3) logistical considerations including commercial airline access, local support from the U.S. Fish and Wildlife Service, and relatively high use by fall sport hunters, all of which facilitate logistically efficient sampling. Viral isolates recovered from sampling at Izembek NWR have led to several important findings regarding intercontinental viral dispersal and evolution. For example, the detection of IAVs at Izembek NWR in 2011 that shared > 99% genomic identity to those of wild bird isolates recovered in China and South Korea supports the intercontinental dispersal of viruses by wild birds among these regions [9]. Additionally, common ancestry of gene segments in the initial H5N2 intercontinental group A (icA) HP reassortant IAV identified in North America in November 2014 with those of viruses recovered from wild birds sampled at Izembek NWR in autumn of the same year is congruent with the hypothesis that migratory birds introduced clade 2.3.4.4 HP IAVs into North America via western Alaska in 2014 and that icA HP reassortant viruses may have first emerged in Beringia [10].

In this study, we evaluate five years of consistent research and surveillance sampling for IAVs in wild birds at Izembek NWR during late summer and early autumn. Specifically, we aim to: (1) evaluate which species and sampling methods are most useful for recovery of IAVs at Izembek NWR, (2) characterize viral subtypes detected in each year and by species to evaluate trends of occurrence, (3) describe genetic diversity for IAVs isolated from wild bird samples collected during 2011–2015, and assess how diversity at this site relates to other regions in North America and East Asia, and (4) evaluate how genetic evidence for intercontinental viral exchange relates to species sampled. Our results will inform future research and surveillance activities at Izembek NWR, provide further inference as to how information from viruses in this region relates to national and international IAV surveillance efforts, and highlight ways by which sampling efficiencies may be identified through critical evaluation of IAV research and surveillance programs.

## Materials and methods

### Sample collection, virus detection, and isolate recovery

During September and October of 2011–2015, and into November in 2013, samples were collected from wild birds within and around Izembek NWR, spatially proximate to the town of Cold Bay, Alaska (55.2045° N, 162.7184° W). Adjacent to Izembek NWR is one of the largest contiguous beds of eelgrass (*Zostera marina*) in the world that supports a large variety of migratory waterfowl and other aquatic birds [11], particularly in the autumn when nearly the entire Pacific flyway population black brant (*Branta bernicla nigricans*) stage in the greater Izembek area [12, 13]. During 2011–2014, hunter-harvested birds were sampled via the cloaca only; in 2015 oropharyngeal and cloacal swabs were taken and paired within the same tube for each bird. In all five years, environmental samples were taken from fecal material deposited by monospecific flocks of emperor geese and glaucous-winged gulls (*Larus glaucescens*), species for which there was no legal sport harvest during the course of this study. Handling of hunter-harvested birds and the collection of fecal samples from the environment does not require institutional animal care and use committee approval. All swab samples were deposited into viral transport media and placed in dry shippers charged with liquid nitrogen within 24 hours. Samples were shipped and stored frozen at -80°C prior to laboratory analysis. Samples collected in 2011–2014 were analyzed at the U.S. Geological Survey National Wildlife Health Center, in Madison, Wisconsin and sample collection efforts and results of virus isolation have been previously reported [9, 10]. Samples collected in 2015 were processed at the Southeastern Cooperative Wildlife Disease Study at the University of Georgia in Athens, Georgia and results are presented here for the first time. Comprehensive summary of viral screening and isolation results from 2011–2015, subtypic and genomic data for five years of isolates recovered, and phylogenetic characterization of all resultant viruses are also presented here for the first time. All samples were screened for the influenza MA gene using real time RT-PCR [14] and those providing cycle threshold values ≤ 45 were inoculated into embryonated eggs for virus isolation [15]. IAV RNA was extracted from virus isolation positive (VI+) samples using MagMax AI/NDV RNA extraction kit (Ambion Inc.) for samples collected 2011–2014, and Qiagen viral RNA mini kit (Qiagen Inc.) for the samples from 2015. All data that support the findings of this publication can be found in Reeves et al. [16] https://doi.org/10.5066/F7JD4W2W.

### Genome sequence generation and subtype designation

IAV genomes were amplified from extracted RNA at the USGS ASC, in a multiplex RT-PCR using Super Script^™^ III One-Step RT-PCR System with Platinum^®^ Taq High Fidelity (Invitrogen), with 2μl of RNA template and the following primers: MBTuni-12 (ACG-CGT-GAT-CAG-CAA-AAG-CAG-G) at 0.1 μM, MBTuni-12(M) (ACG-CGT-GAT-CAG-CRA-AAG-CAG-G) at 0.1 μM, and MBTuni-13 (ACG-CGT-GAT-CAG-TAG-AAA-CAA-GG) at 0.2 μM. Thermocycling conditions were 42°C for 60 min, 5 cycles of 94°C 15 for sec, 45°C for 30 sec, 68°C for 3 min, and 30 cycles of 94°C for 15 sec, 57°C for 30 sec, and 68°C for 3min. Following product visualization and verification (5μl on a 1.0% agarose gel), excess dNTPs and primers were removed using ExoSAP-IT^®^ (USB Corporation). PCR products were quantified using a Quant-iT dsDNA HS Assay Kit (Invitrogen) and prepared for sequencing using a Nextera XT DNA library preparation kit (Illumina, Inc.). Indexed libraries were pooled and sequenced on the Illumina MiSeq using a 500 cycle reagent kit with paired-end reads. We assembled sequence reads from samples collected 2011–2014 using Bowtie 2 version 2.2.3 [17] and those from 2015 with Geneious 9.1.3 [18] using reference data for IAVs obtained from GenBank [19] to map reads. Sanger sequencing data was generated using specific primers to complete consensus sequences for assemblies with gaps and/or poor coverage resulting in low alignment quality scores, as well as validating ambiguities in sequences of suspected mixed infections. Consensus sequences were verified and surface glycoproteins subtypes were verified using FLuANotation (FLAN; Bao, Bolotov [20]). Subtypes were summarized by host species group (dabbling ducks, sea ducks, emperor goose, and glaucous-winged gull) and year. Host species groups used to summarize subtype diversity differed from taxonomic groups used to describe genetic diversity and to assess evidence for intercontinental virus exchange as described subsequently. GenBank accession numbers for viruses isolated as part of this study are: KP336376–KP336391, KT338310–KT338613, KY130518–KY131191, and KY131247–KY131435.

### Viral characterization through phylogenetic analyses

To obtain information on the diversity of IAVs at Izembek NWR and to assess evidence for intercontinental viral exchange, we constructed phylogenies including all available sequence information for isolates originating from wild bird samples collected in Asia and North America over the period of this study as reported by the National Center for Biotechnology Information (NCBI). These reference avian IAV sequences were queried and downloaded on November 7, 2016 using the NCBI Influenza Virus Database https://www.ncbi.nlm.nih.gov/genomes/FLU/Database/nph-select.cgi and the following parameters: Host = Avian; Country/Region = Asia and North America; Collection date = 2011–2015, Sequence length = Full-length only; Collapse identical sequences = filter on. Downloaded files were manually filtered to remove sequences from probable domestic hosts along with those with for which wild or domestic status could not be determined. Specifically, we removed sequences with the following host names: goose, duck, muscovy, ruddy shelduck, turkey, chicken, pheasant, quail, guinea fowl, peacock, and pigeon. Sequences from this study and those downloaded from GenBank were then imported into Geneious 9.1.3 where they were aligned using MUSCLE [21] and cropped to the complete coding region for each of eight gene segments (PB2, PB1, PA, HA, NP, NA, MA, and NS). Maximum likelihood phylogenetic trees were then created using genetic information for each gene segment in MEGA 7.0.21 with the model of the highest log likelihood as tested in MEGA (General Time Reversible model; GTR) and bootstrapping test with 500 replications.

**Fig 1:**
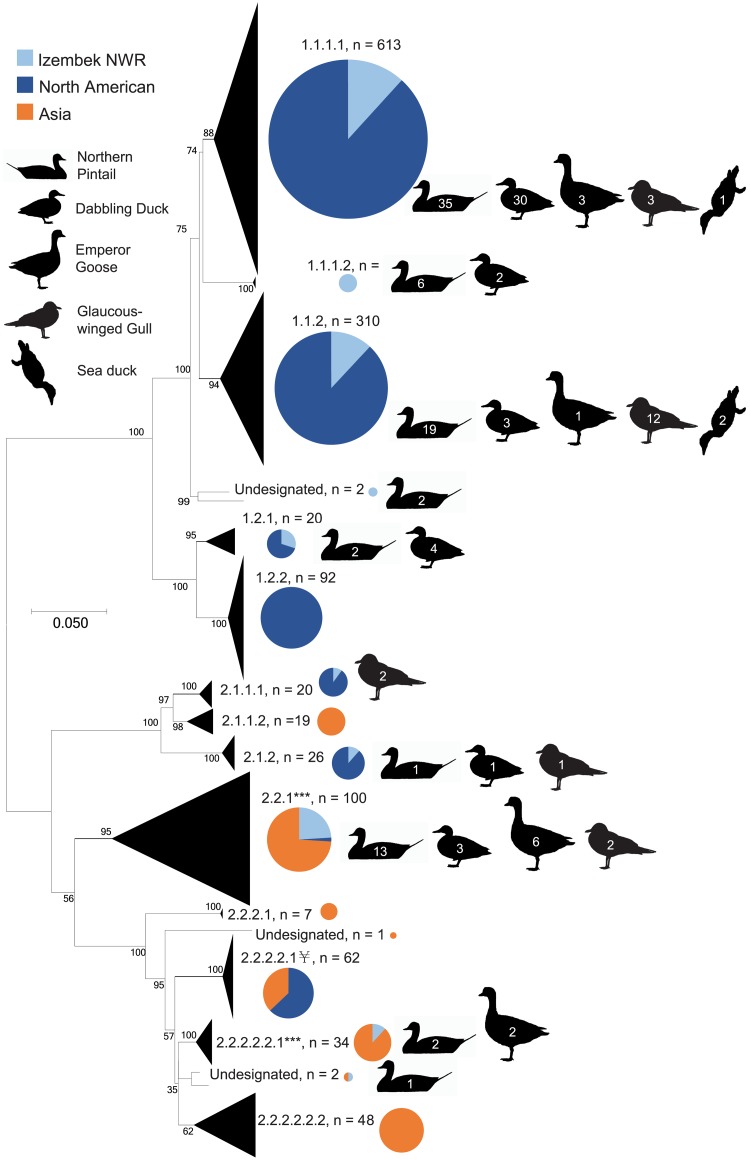
Phylogeny of PB2 nucleotide sequences. The Maximum Likelihood phylogeny of PB2 nucleotide sequences originating from wild bird samples collected in Asia and North America (2011–2015) including those collected from Izembek National Wildlife Refuge (NWR) and sequenced as a part of this study. This phylogeny illustrates the delineation/naming of clades for the purpose of assessing diversity of viruses across host species and the identification of lineages for which there is evidence of intercontinental viral exchange. Clades are collapsed for visualization purposes; the height of collapsed clades (triangles) 1.1.1.1 and 1.1.2 are not proportional to the rest of the phylogeny and instead reduced for presentation purposes. Pie charts at right of clades are proportional in size to the total number of sequences within each clade and color-coded based on the geographical origins of sequences: Izembek NWR = light blue, elsewhere in North America = dark blue, Asia = orange. Clades 2.2.1 and 2.2.2.2.2.1, marked with ‘***,’ are examples of clades providing evidence of intercontinental dispersal of viruses (i.e. designated ‘Asian/mixed origin” via our methods). Clade 2.2.2.2.1, marked with ‘¥,’ comprised of 62 sequence from Asia and North America associated with recent outbreak of highly pathogenic H5 intercontinental group clade A viruses. Sequences identified as “undesignated” did not meet minimum criteria for clade designations (e.g. ≥ 4 sequences). Sequences originating from Izembek NWR in each clade are represented with silhouettes to the right of pie charts corresponding to the species/group from which isolates were derived as used in our data summary and analyses (northern pintail, other dabbling duck, emperor goose, glaucous winged gulls, and sea duck; as described in our methods, several analyses omitted data from viruses isolated from sea ducks). Numbers overlaid on silhouettes provide the number of viral sequences from Izembek NWR for each host species group per clade. For the complete PB2 phylogeny with individual strain names see S1 Fig.

To describe genetic diversity of IAVs detected in wild birds inhabiting North America and Asia, we defined clades for each phylogeny using the following criteria: (1) a clade must contain ≥ 4 strains and be supported by a bootstrap (BS) ≥ 60 with a between-clade nucleotide pairwise distance (PD; calculated with MEGA) ≥ 0.050, or (2) have BS ≥ 95 and PD ≥ 0.040, to the closest neighboring clade meeting equivalent criteria. If a sequence could not be assigned to a clade meeting these criteria we identified it as ‘undesignated’ and excluded the data from further genetic characterization. Clades were designated numerically from top to bottom and from nodes to tips based on furcations in the original tree topology with periods representing nodes (see example, Fig 1). Clade nomenclature used to designate lineages in this study does not follow any previous attempts to classify IAV lineages, nor do we imply any intent for its use in future studies. To evaluate how host species sampled at Izembek NWR may be associated with evidence of intercontinental exchange of IAVs, we identified all clades with sequences originating from Izembek NWR and tabulated the characteristics of each clade based on host species and continental-origins of the sequences within. For the purposes of this study, a clade including only Izembek NWR origin IAV sequences was defined as “Izembek-origin.” Clades including Izembek NWR and other North American sequences were considered “North American-origin.” Lastly, if a clade included sequences from Izembek NWR and any sequences from Asia, the clade was labeled “Asian/mixed-origin.” Izembek-origin clades are characterized in the results and the sequences within them are included in the tests of independence for overall diversity across the phylogenies, however the data from Izembek-origin clades was not used in the analyses assessing for evidence of intercontinental viral exchange as described below.

### Statistical tests of independence

#### Assessing viral diversity by taxa

To evaluate differences in genetic diversity of virus isolates among wild ducks, geese and gulls sampled at Izembek NWR, we calculated how viral sequences originating from host groups were distributed across phylogenetic clades. First, we designated IAV hosts sampled at Izembek NWR into four groups *a priori*: 1) northern pintail, 2) other dabbling duck (American green-winged teal [*Anas crecca*], mallard [*A*. *platyrhynchos*], and northern shoveler [*A*. *clypeata*]), 3) emperor goose and 4) glaucous-winged gull. Northern pintail samples were treated independently from other dabbling duck samples from Izembek NWR due to the intercontinental migratory tendencies of this species (Miller et al., 2005; Hupp et al., 2011), an attribute that we wanted to gain further inference into relative to IAV diversity and evidence for intercontinental viral exchange. Furthermore, all other species of dabbling ducks were combined given comparatively small sample sizes obtained for each species during the course of our study and previously published evidence suggesting that American green-winged teal, mallard, and northern shoveler have less of a tendency to disperse intercontinentally as compared to northern pintails [22, 23]. We did not include virus sequences from the single king eider (*Somateria spectabilis*) isolate nor three isolates originating from bufflehead (*Bucephala albeola*) in any of our statistical analyses given that they represent a small sample for sea ducks as a host group and these hosts did not align taxonomically within any of our other four host species groups. We used Fisher’s exact tests of independence to assess statistical associations between host species groups and the distribution of our IAV sequences among clades for each gene segment. Eight tests (one for each gene segment) were completed for six pairwise comparisons of sequences among four host groups (i.e., ^1^northern pintail to dabbling duck, ^2^northern pintail to emperor goose, ^3^northern pintail to glaucous-winged gull, ^4^dabbling duck to emperor goose, ^5^dabbling duck to glaucous-winged gull, ^6^emperor goose to glaucous-winged gull). We applied a Bonferroni correction for the six pairwise comparisons at each segment, and therefore interpreted p-values < 0.0083 to be significant.

#### Evaluating evidence of intercontinental viral exchange

To evaluate if different wild bird hosts sampled at Izembek NWR were differentially associated with genetic evidence of intercontinental IAV exchange, we evaluated genetic viral diversity relative to previously defined host groups. However, for this analysis, we compared the proportion of IAV gene segment sequences for isolates derived from samples collected at Izembek NWR in Asian/mixed-origin and North American-origin clades by host group. We inferred that the proportion of viral sequences in phylogenetic clades of Asian/mixed-origin clades is useful for evaluating evidence for intercontinental viral exchange although we made no inference regarding directionality of viral dispersal or have meant to imply that intercontinental introduction events occurred at Izembek NWR. Thus, Fisher’s exact tests of independence were used to compare the proportion of IAV sequences in Asian/mixed-origin clades vs. North American-origin clades among previously defined host groups sampled at Izembek NWR (northern pintail, other dabbling ducks, emperor goose, and glaucous-winged gull) where data for all gene segments were combined. Thus, we made six pairwise statistical comparisons among four host groups; we applied a Bonferroni correction and therefore considered p-values < 0.0083 to be significant.

## Results

### Isolate recovery and subtype distribution by year and species sampled

A total of 4842 samples were collected at Izembek NWR from 25 wild bird species over five years (Table 1). The apparent IAV prevalence (MA+ samples / total samples) for all years among commonly harvested dabbling ducks (*Anas spp*.) was similar (Table 1; 5-year totals not shown): northern pintail, 218/1054 (21%); American green-winged teal 66/250 (26%); mallard 37/157 (24%). However, in *Anas* ducks with smaller sample sizes apparent IAV prevalence was lower or zero: northern shoveler, 4/26 (15%); American wigeon (*A*. *americana*), 1/47 (2%); Eurasian wigeon (*A*. *penelope*), 0/19; and gadwall (*A*. *strepera*), 0/15. The 5-year IAV prevalence for hunter-harvested geese species sampled (i.e. cackling goose (*Branta bernicla*) 22/321 [7%], and black brant (*Branta hutchinsii*) 6/313 [2%]) was lower compared to commonly sampled dabbling ducks (northern pintail, American green-winged teal, and mallard). MA+ samples were also detected in king eider 2/5 (40%), bufflehead 5/25 (20%), greater scaup (*Aythya marila*) 1/60 (2%) and long-tailed duck (*Clangula hyemalis*) 1/16 (6%). For fecal samples, the proportion of MA+ to total samples collected from glaucous-winged gulls and emperor geese was 69/1256 (5%) and 77/1159 (7%) respectively. All samples from 12 additional species yielded no MA+ results (Table 1).

**Table 1:** Summary of surveillance sampling of wild birds for influenza A viruses at Izembek National Wildlife Refuge, Alaska, USA 2011–2015.

Species	2011^†^			2012^†^			2013^†^			2014^†^			2015^‡^		
	n =	MA+	VI+	n =	MA+	VI+	n =	MA+	VI+	n =	MA+	VI+	n =	MA+	VI+
Glaucous-winged gull* (*Larus glucescens*)	152	6 (4%)	3	302	6 (2%)	1	253	22 (9%)	12	346	6 (2%)	3	203	29 (14%)	1
Emperor goose* (*Chen canagica*)	99	10 (10%)	2	298	10 (3%)	3	263	5 (2%)	1	292	13 (4%)	3	207	39 (19%)	4
Northern pintail (*Anas acuta*)	226	38 (17%)	25	245	34 (14%)	16	237	32 (14%)	11	147	40 (27%)	17	199	74 (37%)	18
American green-winged teal (*Anas crecca*)	47	15 (32%)	6	31	4 (13%)	3	33	7 (21%)	1	69	17 (25%)	8	70	23 (33%)	10
Mallard (*Anas platyrhynchos*)	18	1 (6%)	0	22	3 (14%)	2	32	3 (9%)	2	49	18 (37%)	8	36	12 (33%)	6
Bufflehead (*Bucephala albeola*)	nt	nt	nt	11	1 (9%)	1	4	2 (50%)	0	10	2 (20%)	2	nt	nt	nt
Northern shoveler (*Anas clypeata*)	1	1 (100%)	0	2	0	0	2	0	0	5	1 (20%)	0	16	2 (13%)	2
King eider (*Somateria spectabilis*)	nt	nt	nt	nt	nt	nt	5	2 (40%)	1	nt	nt	nt	nt	nt	nt
Cackling goose (*Branta hutchinsii*)	220	5 (2%)	0	nt	nt	nt	1	0	0	nt	nt	nt	100	17 (17%)	0
Black brant (*Branta bernicla*)	191	0	0	nt	nt	nt	1	0	0	21	0	0	100	6 (6%)	0
Greater scaup (*Aythya marila*)	15	0	0	13	0	0	8	1 (13%)	0	21	0	0	3	0	0
American wigeon (*Anas americana*)	17	0	0	13	0	0	4	0	0	11	1 (9%)	0	2	0	0
Long-tailed duck (*Clangula hyemalis*)	nt	nt	nt	3	0	0	13	1 (8%)	0	nt	nt	nt	nt	nt	nt

Abbreviations have been used for number of samples collected (n =), matrix positive samples (MA+), and virus isolation positive samples (VI+). Results from 2011–2014 were previously summarized by Ramey et al. [9, 10]. Minor discrepancies between results reported here and previous summaries are on account of methodological differences between studies (e.g. cloacal or cloacal/oropharyngeal samples collected from deceased Glaucous-winged gulls [n = 5] and Emperor geese [n = 5] were omitted from this study). The following species and the corresponding total number samples taken across all years were not included in Table 1 as a result of negative rRT-PCR results targeting the matrix gene for all samples: Harlequin duck (*Histrionicus histrionicus*; n = 50), Common eider (*Somateria mollissima*; n = 21), Eurasian wigeon (*Anas penelope*; n = 19), White-winged scoter (*Melanitta fusca*; n = 18), Gadwall (*Anas strepera*; n = 15), Red-breasted merganser (*Mergus serrator*; n = 10), Black scoter (*Melanitta americana*; n = 10), Common goldeneye (*Bucephala clangula*; n = 4), Greater white-fronted goose (*Anser albifrons*; n = 2), Lesser scaup (*Aythya affinis*; n = 2), Ring-necked duck (*Aythya collaris*; n = 1), and Canvasback (*Aythya valisineria*; n = 1)

*Environmental samples collected from feces of visually identified monospecific flocks

^†^Cloacal samples from hunter-harvested birds 2011–2014; rRT-PCR and VI laboratory work completed at U.S. Geological Survey National Wildlife Health Center

^‡^Cloacal and oropharyngeal samples pooled for each hunter-harvested bird 2015; rRT-PCR and VI laboratory work completed at UGA Southeast Cooperative Wildlife Disease Study

In total, 172 viral isolates (average: 34.4 isolates/year; range: 26–41) were identified and genomically sequenced (Table 1). All isolates were predicted to be of low pathogenicity in poultry given the lack of a polybasic cleavage site in the hemagglutinin gene segments. Just over half of the isolates (n = 87) came from northern pintails samples; the remaining, in descending numerical order, were collected from American green-winged teal (n = 28), glaucous-winged gull (n = 20), mallard (n = 18), emperor goose (n = 13), bufflehead (n = 3), northern shoveler (n = 2), and king eider (n = 1; Table 1). Isolates were recovered in all five years of this study from northern pintail, American green-winged teal, emperor goose, and glaucous-winged gull. No isolates were recovered from 17 additional waterfowl species, including cackling goose and black brant where samples sizes exceeded 300 (each) over the five year study period (Table 1).

Seven isolates that were identified as mixed infection produced two sequences each for the HA and NA segments [16]. For these isolates, both HA and both NA subtypes were included in our assessment of subtype frequencies by species and by year, however HA-NA combinations were not inferred from mixed infections (Table 2). When considering IAV subtypes by species groups, H3 and N8 were the most common HA and NA subtypes, respectively, for dabbling duck (*Anas spp*.) and Emperor goose isolates across all years with the H3N8 subtype combination being most common for both taxonomic groups (37% of dabbling duck isolates and 46% of emperor goose isolates; [16]). The second most abundant subtype combination for isolates derived from these species across the 2011–2015 sampling period was H4N6 (15% of dabbling duck isolates and 15% of emperor goose isolates). In comparison, the HA H13 and NA N2 subtypes were the most common observed from glaucous-winged gull isolates during 2011–2015, with the H13N2 subtype combination being most common (50% of all glaucous-winged gull isolates) followed equally by H4N6 and H16N3 (10% of all glaucous-winged gull isolates for each combined subtype). Three of the four isolates from sea ducks (bufflehead and king eider) were of the H4 HA subtype, the fourth was an H7, however and all four NA sequences were of different serotypes (N2, N4, N6, and N8).

**Table 2:** Summary of subtype combinations detected from IAV isolates originating from samples collected at Izembek National Wildlife Refuge in Alaska, USA 2011–2015.

Subtype	2011	2012	2013	2014	2015	total	Host Taxon
H1N1				5		5	G, D
H2N3	2					2	D
H2N9	1					1	D
H3N1				1		1	D
H3N3				1		1	D
H3N6	1			1	3	5	D
H3N8	12	19	6	6	14	57	G, E, D
H4N1				1		1	D
H4N2		1				1	D
H4N6	4	3	4	7	7	25	G, E, D, S
H4N8				1		1	S
H5N2	1	1		1		3	G, D
H5N3				1		1	D
H6N1					5	5	D
H6N2	2				1	3	D
H6N6			1			1	D
H6N8			2		1	3	D
H7N3			1			1	D
H7N4				3		3	D, S
H8N4	1				1	2	D
H8N8	1					1	D
H9N2	2					2	E, D
H10N7		1	1		2	4	E, D
H11N2				2		2	G, E
H12N5	1					1	D
H13N2			10			10	G
H16N3			1	3		4	G, D
mixed	8	1	2	8	7	26	G, E, D, S

All isolates with more than one genetic sequence detected for one or more gene segment are reported here as “mixed” infection. D = dabbling duck (*Anas acuta*, *A*. *crecca*, *A*. *platyrhychos*, and *A*. *clypeata*; n = 135), E = emperor goose (*Chen canagica*; n = 13), G = glaucous-winged gull (*Larus glucescens*; n = 20), and S = sea duck (i.e. *Bucephala albeola* and *Somateria spectabilis*; n = 4).

**Fig 2:**
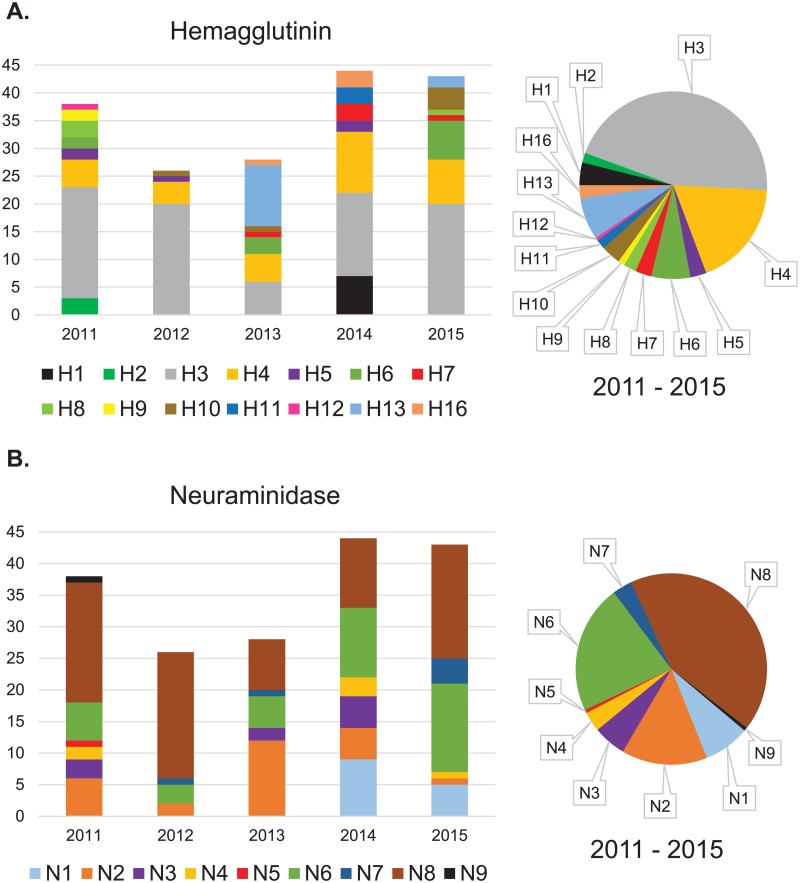
Hemagglutinin and Neuraminidase subtypes identified by year. Totals and proportions of influenza A virus subtypes (Hemagglutinin [HA, A]; Neuraminidase [NA, B]) identified per year from virus isolation positive samples collected at Izembek National Wildlife Refuge, Alaska, USA. The number of sequences identified for each of the surface glycoproteins are 38, 26, 28, 44, and 43 for the years 2011–2015, respectively. The total numbers of HA and NA sequences (179 each) exceed the total number of isolates genomically sequenced (172) because two HA and two NA sequences were identified from seven mixed infection samples.

In total, 26 different viral subtype combinations were identified (mixed infection data not included) from H1–H13, H16 and N1–N9 surface glycoprotein sequences (Table 2). H3 was the most common HA subtype in all years except 2013 during which H13 was more common (Fig 2). In that year (2013) viruses of the H13 subtype (n = 12) were only found in glaucous-winged gulls [16]. N8 was the most frequently observed NA subtype for all years except for 2013 when N2 was more common (Fig 2). Correspondingly in 2013, all 12 H13 isolates were of the H13N2 combined subtype. The most frequently observed subtype combination for years 2011, 2012, and 2015 was H3N8. In 2013 H13N2 was the most frequently detected subtype combination, as was H4N6 for sample year 2014 (Table 2). H3N8 and H4N6 were the only subtype combinations detected in all five years of this study.

### Genetic diversity of 2011–2015 Izembek NWR IAV isolates by species group

We made a single exception to our *a priori* clade designation criteria (see Methods) by designating MA clade 1.1.1.1 which was supported by a bootstrap value < 60 (specifically 52). Using our *a prioi* criteria, this clade should have been undesignated with sequences not included in statistical tests of diversity, nor in tests assessing genetic evidence for intercontinental IAV exchange. However, the majority of the MA sequences (810 of 1064 sequences, including 133 of 157 for our Izembek NWR isolates; S5 Fig) were nested within this clade, and therefore omitting this data from statistical tests (i.e., by considering this clade undesignated) would have potentially biased results by using only a minority of sequence information for this gene segment (i.e., those sequences which clustered within smaller diverse clades). As such, the number of clades designated per gene segment using our *a priori* criteria and modified to include this single exception for the MA gene segment, ranged from 9–17 (average = 13.8) for the internal segments, and 1–9 (average = 3.8) for HA and NA phylogenies (S1–S29 Figs). Although we identified H8 sequences in isolates obtained from Izembek NWR, and information was obtained from NCBI for gene segments of this subtype, the NCBI GenBank database contained too few sequences (i.e. North America, n = 4; Asia, n = 0) over the same time period to create an informative phylogeny for the purposes of this study. Information for the H8 HA gene segment (S15 Fig) was therefore excluded from further characterization.

We identified 92 total clades among our phylogenies for all gene segments that included sequences for IAV isolates from wild bird hosts sampled at Izembek NWR. Two clades included only sequences from Izembek NWR. There were eight Izembek NWR IAV sequences from dabbling duck samples collected in 2011 in PB2 clade 1.1.1.2, (six northern pintail and two American green-winged teal isolates; Fig 1 and S1 Fig). This Izembek-origin clade was 6.4% divergent at the nucleotide level from the most closely-related sister PB2 clade 1.1.1.1, a large clade of North American-origin sequences including 72 from Izembek NWR. Additionally, H13 clade 1.1.1 was comprised of 11 closely-related sequences originating from glaucous-winged gulls collected at Izembek NWR in 2013 (S20 Fig). This clade was 8.8% divergent at the nucleotide level from sister clade 1.1.2 which was found in multiple gull species sampled in both Asia (i.e. Republic of Georgia, n = 5), and North America (i.e. Massachusetts, n = 1; and Izembek NWR, n = 1). Of the 92 phylogenetic clades designated as part of this study that included sequences for Izembek NWR isolates, approximately two-thirds (n = 61) included sequences originating from more than one host species group (i.e., northern pintail, other dabbling duck, emperor goose, or glaucous-winged gull). The remaining 31 clades included Izembek NWR IAV sequences from only northern pintail (n = 13), other dabbling duck (n = 6) or glaucous-winged gull (n = 12) hosts. There were no clades designated in our study that included emperor goose viral sequences without also including Izembek NWR IAV sequences from another host species group.

The majority of the 92 clades that included viral gene segment sequences for IAVs from Izembek NWR (n = 73) included northern pintail origin isolates or those from other dabbling ducks (n = 58). A minority of clades contained sequences for isolates from emperor goose (n = 34) or glaucous-winged gull (n = 44). When assessing the distribution of IAV sequences across clades, and accounting for sample size differences across host groups, viral sequences from glaucous-winged gulls were significantly different in their distribution across clades for six of eight segments as compared to those originating in northern pintails, to other dabbling ducks, and to emperor geese (two-tailed Fisher’s exact test, glaucous-winged gulls vs. [each] northern pintails, other dabbling ducks, and to emperor geese at [each] PB2, PA, HA, NP, NA, and NS; p-values < 0.0083, S1 Table). However, the distributions of IAV sequences across clades for northern pintail, other dabbling duck and emperor goose hosts were not significantly different when compared among one another, except for one of eight gene segments for each set of pairwise comparisons (two-tailed Fisher’s exact tests: northern pintail vs other dabbling ducks at the HA gene segment, p-value = 0.0016; northern pintail vs emperor goose at the PA gene segment, p-value = 0.0078; and other dabbling duck vs emperor goose at the PB2 gene segment, p-value = 0.0008; S1 Table).

### Assessing evidence of intercontinental viral exchange among host groups

The proportion of viral sequences from Izembek NWR isolates that were assigned to clades designated as Asian/mixed-origin by species group were: 108 of 651 (16.6%) for northern pintail, 30 of 355 (8.5%) for other dabbling duck, 20 of 101 (19.8%) for emperor goose, and 47 of 153 (30.7%) for glaucous-winged gull. A significantly lower proportion of sequences for IAVs recovered from other dabbling ducks were found to be in Asian/mixed-origin clades as compared to northern pintail, emperor goose, and glaucous-winged gull host species groups (two-tailed Fisher’s exact tests: other dabbling ducks vs. northern pintail, p-value = 0.0009; other dabbling ducks vs emperor goose, p-value = 0.0033; and other dabbling duck vs glaucous-winged gull, p-value < 0.0001; S1 Table). Sequences for IAVs from glaucous-winged gull were also significantly more likely to be in Asian/mixed-origin clades as compared to northern pintail (other northern pintail vs glaucous-winged gull, p-value < 0.0001).

## Discussion

### Identifying sampling strategies useful for IAV isolate recovery

Through the collection of samples from wild birds at Izembek NWR over five years, we were able to consistently detect and isolate IAVs from hunter-harvested dabbling ducks, particularly northern pintails, the most abundant and commonly harvest dabbling duck at Izembek NWR in late summer and autumn, and from glaucous-winged gull and emperor goose feces collected from the environment. While numerous studies in Alaska and elsewhere have reported success in isolating IAVs from northern pintail and glaucous-winged gull [2, 4, 24, 25], we are unaware of other locations in Alaska or Russia where sampling of emperor geese has consistently resulted in isolation of IAVs. We suspect the relative abundance of IAV-naïve hatch-year emperor geese at this location during autumn contributed to an increased probability of viral isolation from this population. Therefore, Izembek NWR may represent a useful location for targeted sampling of this species. We also isolated four additional viruses from two sea duck species, king eider and bufflehead, however, sample sizes were smaller for this taxonomic group as these species are less-commonly targeted by hunters at Izembek NWR during September–October. Thus a lack of availability makes these species poor targets for IAV research and surveillance at this site during late summer and autumn. We were unable to isolate viruses from hunter-harvested cackling goose and black brant during the course of this study, with the caveat that these geese were not sampled consistently from year to year. While collecting additional hunter-harvested samples from these species at Izembek NWR during September–October is feasible, previous sampling of cackling goose and black brant in Alaska suggests that the probability of successful virus isolation is low (i.e., < 0.01) and that IAVs may be most likely recovered from samples collected from these species during spring [5, 26]. Thus, in the absence of rationale for targeted sampling of cackling geese and black brant, there appears to be poor support for routine sampling of these species for IAVs in future research and surveillance efforts conducted at Izembek NWR in late summer and autumn.

### Subtypes and genetic diversity of IAVs from wild birds sampled at Izembek NWR

The two most common IAV HA subtypes detected in waterfowl at Izembek NWR in 2011–2015 were H3 and H4, consistent with results for waterfowl sampled elsewhere in Alaska [4, 5, 22] and in North America [27–31] where H3 and H4 IAVs were common. We detected the NA subtypes N6 and N8 most commonly from waterfowl over the study period, and congruent with HA results, these serotypes are also reported as common in other North American studies in Alaska [4, 5, 22] and elsewhere [27, 28, 31]. Additionally, the most common HA subtype in samples collected from glaucous-winged gulls was H13. While most of the H13 viruses were isolated from gull samples from a single year (2013), the result is consistent with previous studies reporting an association between H13 viruses and gull species throughout the Northern Hemisphere [32–36]. Other research and surveillance efforts targeting gulls have reported the H16 HA subtype to be common [37, 38] and we detected two isolates of this subtype from glaucous-winged gull samples collected in 2013 and 2014, as well as an isolate each from a northern pintail and a mallard in 2014. Results of a *post hoc* query of the Influenza Virus Resource [19] on December 11, 2017 support a strong association of IAVs of the H16 subtype with gulls, with 250 of 270 sequences (93%; including sequences from this study) for this subtype being reported in gulls while only 7 of 270 sequences (3%) originated from samples collected from ducks. In this study, all HA H13 and H16 sequences (excluding isolates identified as mixed infections) were paired with NA N2 and N3 respectively, a finding that was not surprising considering the frequently observed association between HA and NA subtypes in the H13N2 and H16N3 combinations, particularly for IAVs fromgulls sampled in North America [32, 38] and Europe [35, 39]. Thus, sampling gulls and waterfowl at Izembek NWR appears to provide slightly different information regarding the circulation of IAV subtypes at this site, consistent with the idea that multiple, yet overlapping, IAV gene pools are maintained among these taxa [40].

When we compared the genetic diversity of viruses recovered from samples collected at Izembek NWR during 2011–2015 as compared to reference sequences reported on GenBank from Asia and elsewhere in North America, we found that some clades for the PB2 and H13 HA gene segments were exclusive to sequences originating from Izembek NWR. Results for *post hoc* BLAST analysis for the Izembek-origin clade PB2 1.1.1.2 show these viruses share 98–99% nucleotide identity to viruses collected from wild birds in Alaska and western North America prior to 2011. Additionally, BLAST results for the Izembek-origin clade H13 1.1.1 shared 98% nucleotide identity to black-headed gull (*Chroicocephalus ridibundus*) isolates recovered in Europe in years up to and including 2011. Thus, it appears that previously identified IAV lineages are genetically similar to our sequences within ‘Izembek-origin’ clades, however not during concurrent sampling efforts in adjacent regions. This finding suggests that conducting research and surveillance for IAVs at Izembek NWR may yield genetic information not readily found in concurrent sampling programs in Asia or elsewhere in North America or that there are biases in online public databases containing genetic information for wild bird-origin IAVs.

When we examined genetic diversity of IAVs recovered from sampling efforts conducted at Izembek NWR during 2011–2015 relative to species sampled, we did not detect significant differences in genetic diversity in pairwise tests among northern pintail, other dabbling duck and emperor goose for most IAV gene segments. In contrast, the genetic diversity of IAVs isolated from glaucous-winged gulls was generally different from these waterfowl host species groups. Similar to our results comparing subtypes by taxa, and congruent with evidence of somewhat different but overlapping gene pools for gulls and waterfowl [40], we garnered greater viral diversity information through the sampling of both glaucous-winged gulls and waterfowl at this site. This information helps optimize surveillance efforts from a sampling-efficiency standpoint. USGS ASC research and surveillance efforts at Izembek NWR have relied on sampling of feces from the environment to obtain viral information from glaucous-winged gulls given that this species is not open to sport harvest. The IAV isolation rates from fecal samples were consistently lower compared to the hunter-harvested waterfowl samples. This difference may be the result of sample type, host species sampled, or a combination of the two. Regardless, a lower IAV isolation rate for environmental fecal samples translates into an increase in the number of samples necessary to collect and screen per virus obtained. Thus, this study provides evidence that, while the number of isolates per sample collected and screened may be lower for species such as northern pintail or other dabbling ducks, different information on viral diversity maintained in wild birds at Izembek NWR is obtained by sampling feces from glaucous-winged gulls as compared to waterfowl. In contrast to this finding, clades of IAVs were unique to all species groups included in our analysis of samples collected at Izembek NWR during 2011–2015, except for emperor goose. Emperor geese were closed to sport harvest during our five year study and we relied on environmental fecal sampling to recover isolates from this taxon, similar to glaucous-winged gulls. Given that our results suggest that comparable information regarding viral diversity may be obtained from sampling of hunter-harvested northern pintail and other dabbling ducks as compared to emperor goose, screening comparatively larger numbers of samples per IAV isolate recovered from emperor goose fecal material may not be as efficient as sampling hunter-harvested waterfowl in late summer and autumn at Izembek NWR. However, one limitation to IAV research and surveillance at a remote location such as Izembek NWR is that the relatively small number of sport hunters harvesting birds during late summer and autumn limits the number of ducks that can be sampled. During our annual late summer and autumn sampling period we collected swab samples from every hunter-harvested duck (dabbling, diver and sea duck) available to us resulting in a peak of 426 ducks sampled in 2013 [16]. Therefore increasing the sample size for swabs from hunter-harvested northern pintails and other dabbling ducks will be dependent on some combination of: more hunters, greater harvest of duck, and/or increased participation of hunters to provide ducks for sampling. Environmental fecal sampling, however, is independent of hunter-harvest sampling efforts and can be adjusted in the field as necessary to meet project goals.

### Associating host species with evidence for intercontinental exchange of IAVs

With regard to our assessment of evidence for intercontinental dispersal of IAVs at Izembek NWR relative to host species groups, we found genetic sequences for viruses from northern pintail, emperor goose, and glaucous-winged gulls were more frequently found in Asian/mixed clades compared to IAVs isolated from other dabbling ducks. These findings may be attributed, at least partially, to the intercontinental migratory tendencies for northern pintail and emperor goose relative to other species of North American waterfowl and unidentified mechanisms driving a more cosmopolitan distribution of IAV lineages among North American gulls. First, with regard to waterfowl species sampled, Pearce et al. [22] previously assessed the influence of migratory tendencies and breeding ground sympatry relative to the intercontinental exchange of IAVs in Alaska by comparing the band recovery data for mallards and northern pintails and the detection of Eurasian origin IAV gene segments from isolates recovered through sampling these species in Alaska. The authors found greater evidence for intercontinental viral exchange in northern pintails, a species more commonly associated with migratory flights between North America and East Asia, as compared to mallards, a species for which there is little evidence for intercontinental movement as assessed through band recovery data. However, an important caveat in the study by Pearce et al. [22] is that IAVs analyzed from northern pintails were disproportionately from western Alaska, a region where Eurasian lineage genes have consistently been detected at higher rates as compared to elsewhere in Alaska or North America [4, 5, 41], as compared to the IAVs used to assess evidence for intercontinental virus exchange in mallards which were sampled at higher proportions at interior and southeastern portions of the state. In this study, we compared waterfowl at a single location in Southwestern Alaska and, in agreement with results presented by Pearce et al. [22], we also found a higher proportion of IAVs displaying evidence for intercontinental exchange in waterfowl with inter-hemispheric migratory tendencies (i.e., northern pintail and emperor goose) as compared to other dabbling ducks (i.e. American green-winged teal, mallard, and northern shoveler) which are less intercontinentally vagile [22, 23]. In concurrence with conclusions put forth by Pearce et al. [22], our study also provides evidence that targeted sampling of waterfowl with intercontinental migratory tendencies may be the most efficient method to detect evidence of inter-hemispheric viral exchange. Additional sampling of sympatric ‘sentinel’ species (i.e., those with less of a tendency to make trans-hemispheric movements) at breeding or staging areas, with whom IAVs will likely be exchanged at some level, may also be a useful strategy for detecting evidence for intercontinental IAV exchange, albeit at a lower level, given logistical considerations of research and surveillance efforts.

Regarding the distribution of viral lineages in gulls, previous studies have identified numerous intercontinental reassortant viruses in gulls sampled throughout the Northern Hemisphere [32, 41–44], and found the detection of such viruses to be particularly common in gulls sampled within North America [25]. Intercontinental migratory movements of individuals of some gull species and subsequent viral dissemination among sympatric birds within North America may be one plausible explanation for these findings. However, relatively few ornithological investigations have been conducted to assess migratory pathways for North American gull populations, particularly those from Alaska. Observational data suggest that hundreds of thousands of gulls may make migratory movements between Asia and North America each year [45] and band recovery data provide evidence for trans-Atlantic movements of greater black-backed gulls (*Larus marinus*) banded in Eastern Canada to Europe [44]. The only published data regarding migratory movements of glaucous-winged gulls from Alaska is for individuals marked in the southcentral region of the state. Data published by Hatch et al. [46] suggests that glaucous-winged gulls breeding in southcentral Alaska winter in Southeast Alaska, British Columbia, Washington, Oregon, and California. Thus it remains unclear if our finding of extensive evidence for intercontinental IAV exchange in glaucous-winged gulls sampled at Izembek NWR reflects intercontinental movements of this population of birds or whether they are serving as sentinels of viral exchange. We propose that further ornithological work to understand the migratory pathways for gulls is important for interpreting the extensive evidence for intercontinental IAV exchange in North America for this taxonomic group.

### Conclusions for future research and surveillance

Given our evaluation of five years of sampling for IAVs at Izembek NWR, we conclude by making the following observations and recommendations regarding future research and surveillance efforts at this site: (1) Sampling for IAVs at Izembek NWR during late summer and autumn consistently provides evidence for intercontinental viral exchange regardless of species sampled, but particularly for IAVs recovered from waterfowl with interhemispheric migratory tendencies (i.e., northern pintail and emperor goose) and glaucous-winged gulls. Continued sampling of these species at this location may provide additional information on the introduction of IAVs from East Asia to North America and the exchange of viruses between these adjacent regions. (2) Sampling of both hunter-harvested dabbling ducks and environmental feces deposited by glaucous-winged gulls appears to efficiently provide information on the diversity of IAVs circulating among wild birds inhabiting Izembek NWR. Sampling of emperor geese also consistently provides viral isolates including those displaying evidence of intercontinental viral exchange, but larger numbers of samples are required to collect and screen per virus recovered. Thus, sampling of environmental feces from emperor geese should be considered a lower priority at Izembek NWR in late summer and autumn than other host species groups and future research and surveillance efforts should carefully consider goals and available resources when selecting target species at this sampling site. Finally, (3) sampling of cackling geese and black brant is unlikely to result in isolation of IAVs at Izembek NWR during autumn. Without clear rationale for targeted sampling in these species, efforts to recover viruses from cackling geese and black brant at Izembek NWR should be suspended. We encourage other researchers investigating IAVs in wild birds in North America and elsewhere to develop similar evaluations of their research and sampling efforts to maximize efficiencies of the global IAV surveillance system.

## Supporting information

S1 Table Results of Fisher’s exact test of independence comparing influenza A virus (IAV) sequence distributions across all phylogenetic clades and those containing Asian-origin sequences by host species group. Information below the diagonal line of shaded boxes in the table report p-values for comparisons of IAV sequence distributions across all clades (see Methods) within each phylogeny by gene segment. Above the diagonal line of shaded boxes are p-values for comparisons among host species groups with respect to the proportion of IAV sequences in Asian/mixed-origin clades (see Methods). P-values < 0.0083 and therefore interpreted as statistically significant are indicated in bold font.
(DOCX)

S1 Fig Phylogeny of PB2 nucleotide sequences. Maximum likelihood phylogenetic tree based on the General Time Reversible model (G+I) depicting the inferred relationship of the complete coding region of PB2 segment sequences from influenza A viruses collected from wild birds in Asia and North America 2011–2015. A bootstrap analysis was performed with 500 replications; results are displayed at nodes. The analysis involved 1364 nucleotide sequences. All positions containing gaps and missing data were eliminated. There was a total of 2087 positions in the final dataset. Evolutionary analyses were conducted in MEGA7. Sequences originating from Izembek National Wildlife Refuge, Alaska, USA, are identified by asterisks (***) following the strain name. Numerical clade names, provided next to brackets, represent the classification used in this study.
(EPS)

S2 Fig Phylogeny of PB1 nucleotide sequences. Maximum likelihood phylogenetic tree based on the General Time Reversible model (G+I) depicting the inferred relationship of the complete coding region of PB1 segment sequences from influenza A viruses collected from wild birds in Asia and North America 2011–2015. A bootstrap analysis was performed with 500 replications; results are displayed at nodes. The analysis involved 1372 nucleotide sequences. All positions containing gaps and missing data were eliminated. There was a total of 2157 positions in the final dataset. Evolutionary analyses were conducted in MEGA7. Sequences originating from Izembek National Wildlife Refuge, Alaska, USA, are identified by asterisks (***) following the strain name. Numerical clade names, provided next to brackets, represent the classification used in this study.
(EPS)

S3 Fig Phylogeny of PA nucleotide sequences. Maximum likelihood phylogenetic tree based on the General Time Reversible model (G+I) depicting the inferred relationship of the complete coding region of PA segment sequences from influenza A viruses collected from wild birds in Asia and North America 2011–2015. A bootstrap analysis was performed with 500 replications; results are displayed at nodes. The analysis involved 1376 nucleotide sequences. All positions containing gaps and missing data were eliminated. There was a total of 2042 positions in the final dataset. Evolutionary analyses were conducted in MEGA7. Sequences originating from Izembek National Wildlife Refuge, Alaska, USA, are identified by asterisks (***) following the strain name. Numerical clade names, provided next to brackets, represent the classification used in this study.
(EPS)

S4 Fig Phylogeny of NP nucleotide sequences. Maximum likelihood phylogenetic tree based on the General Time Reversible model (G+I) depicting the inferred relationship of the complete coding region of NP segment sequences from influenza A viruses collected from wild birds in Asia and North America 2011–2015. A bootstrap analysis was performed with 500 replications; results are displayed at nodes. The analysis involved 1214 nucleotide sequences. All positions containing gaps and missing data were eliminated. There was a total of 1446 positions in the final dataset. Evolutionary analyses were conducted in MEGA7. Sequences originating from Izembek National Wildlife Refuge, Alaska, USA, are identified by asterisks (***) following the strain name. Numerical clade names, provided next to brackets, represent the classification used in this study.
(EPS)

S5 Fig Phylogeny of MA nucleotide sequences. Maximum likelihood phylogenetic tree based on the General Time Reversible model (G+I) depicting the inferred relationship of the complete coding region of MA segment sequences from influenza A viruses collected from wild birds in Asia and North America 2011–2015. A bootstrap analysis was performed with 500 replications; results are displayed at nodes. The analysis involved 1064 nucleotide sequences. All positions containing gaps and missing data were eliminated. There was a total of 964 positions in the final dataset. Evolutionary analyses were conducted in MEGA7. Sequences originating from Izembek National Wildlife Refuge, Alaska, USA, are identified by asterisks (***) following the strain name. Numerical clade names, provided next to brackets, represent the classification used in this study.
(EPS)

S6 Fig Phylogeny of NS allele A nucleotide sequences. Maximum likelihood phylogenetic tree based on the General Time Reversible model (G+I) depicting the inferred relationship of the complete coding region of NS Allele A segment sequences from influenza A viruses collected from wild birds in Asia and North America 2011–2015. A bootstrap analysis was performed with 500 replications; results are displayed at nodes. The analysis involved 803 nucleotide sequences. All positions containing gaps and missing data were eliminated. There was a total of 784 positions in the final dataset. Evolutionary analyses were conducted in MEGA7. Sequences originating from Izembek National Wildlife Refuge, Alaska, USA, are identified by asterisks (***) following the strain name. Numerical clade names, provided next to brackets, represent the classification used in this study.
(EPS)

S7 Fig Phylogeny of NS allele B nucleotide sequences. Maximum likelihood phylogenetic tree based on the General Time Reversible model (G+I) depicting the inferred relationship of the complete coding region of NS allele B segment sequences from influenza A viruses collected from wild birds in Asia and North America 2011–2015. A bootstrap analysis was performed with 500 replications; results are displayed at nodes. The analysis involved 362 nucleotide sequences. All positions containing gaps and missing data were eliminated. There was a total of 822 positions in the final dataset. Evolutionary analyses were conducted in MEGA7. Sequences originating from Izembek National Wildlife Refuge, Alaska, USA, are identified by asterisks (***) following the strain name. Numerical clade names, provided next to brackets, represent the classification used in this study.
(EPS)

S8 Fig Phylogeny of HA H1 nucleotide sequences. Maximum likelihood phylogenetic tree based on the General Time Reversible model (G+I) depicting the inferred relationship of the complete coding region of HA H1 segment sequences from influenza A viruses collected from wild birds in Asia and North America 2011–2015. A bootstrap analysis was performed with 500 replications; results are displayed at nodes. The analysis involved 79 nucleotide sequences. All positions containing gaps and missing data were eliminated. There was a total of 1682 positions in the final dataset. Evolutionary analyses were conducted in MEGA7. Sequences originating from Izembek National Wildlife Refuge, Alaska, USA, are identified by asterisks (***) following the strain name. Numerical clade names, provided next to brackets, represent the classification used in this study.
(EPS)

S9 Fig Phylogeny of HA H2 nucleotide sequences. Maximum likelihood phylogenetic tree based on the General Time Reversible model (G+I) depicting the inferred relationship of the complete coding region of HA H2 segment sequences from influenza A viruses collected from wild birds in Asia and North America 2011–2015. A bootstrap analysis was performed with 500 replications; results are displayed at nodes. The analysis involved 82 nucleotide sequences. All positions containing gaps and missing data were eliminated. There was a total of 1682 positions in the final dataset. Evolutionary analyses were conducted in MEGA7. Sequences originating from Izembek National Wildlife Refuge, Alaska, USA, are identified by asterisks (***) following the strain name. Numerical clade names, provided next to brackets, represent the classification used in this study.
(EPS)

S10 Fig Phylogeny of HA H3 nucleotide sequences. Maximum likelihood phylogenetic tree based on the General Time Reversible model (G+I) depicting the inferred relationship of the complete coding region of HA H3 segment sequences from influenza A viruses collected from wild birds in Asia and North America 2011–2015. A bootstrap analysis was performed with 500 replications; results are displayed at nodes. The analysis involved 247 nucleotide sequences. All positions containing gaps and missing data were eliminated. There was a total of 1663 positions in the final dataset. Evolutionary analyses were conducted in MEGA7. Sequences originating from Izembek National Wildlife Refuge, Alaska, USA, are identified by asterisks (***) following the strain name. Numerical clade names, provided next to brackets, represent the classification used in this study.
(EPS)

S11 Fig Phylogeny of HA H4 nucleotide sequences. Maximum likelihood phylogenetic tree based on the General Time Reversible model (G+I) depicting the inferred relationship of the complete coding region of HA H4 segment sequences from influenza A viruses collected from wild birds in Asia and North America 2011–2015. A bootstrap analysis was performed with 500 replications; results are displayed at nodes. The analysis involved 272 nucleotide sequences. All positions containing gaps and missing data were eliminated. There was a total of 1680 positions in the final dataset. Evolutionary analyses were conducted in MEGA7. Sequences originating from Izembek National Wildlife Refuge, Alaska, USA, are identified by asterisks (***) following the strain name. Numerical clade names, provided next to brackets, represent the classification used in this study.
(EPS)

S12 Fig Phylogeny of HA H5 nucleotide sequences. Maximum likelihood phylogenetic tree based on the General Time Reversible model (G+I) depicting the inferred relationship of the complete coding region of HA H5 segment sequences from influenza A viruses collected from wild birds in Asia and North America 2011–2015. A bootstrap analysis was performed with 500 replications; results are displayed at nodes. The analysis involved 292 nucleotide sequences. All positions containing gaps and missing data were eliminated. There was a total of 1675 positions in the final dataset. Evolutionary analyses were conducted in MEGA7. Sequences originating from Izembek National Wildlife Refuge, Alaska, USA, are identified by asterisks (***) following the strain name. Numerical clade names, provided next to brackets, represent the classification used in this study.
(EPS)

S13 Fig Phylogeny of HA H6 nucleotide sequences. Maximum likelihood phylogenetic tree based on the General Time Reversible model (G+I) depicting the inferred relationship of the complete coding region of HA H6 segment sequences from influenza A viruses collected from wild birds in Asia and North America 2011–2015. A bootstrap analysis was performed with 500 replications; results are displayed at nodes. The analysis involved 70 nucleotide sequences. All positions containing gaps and missing data were eliminated. There was a total of 1689 positions in the final dataset. Evolutionary analyses were conducted in MEGA7. Sequences originating from Izembek National Wildlife Refuge, Alaska, USA, are identified by asterisks (***) following the strain name. Numerical clade names, provided next to brackets, represent the classification used in this study.
(EPS)

S14 Fig Phylogeny of HA H7 nucleotide sequences. Maximum likelihood phylogenetic tree based on the General Time Reversible model (G+I) depicting the inferred relationship of the complete coding region of HA H7 segment sequences from influenza A viruses collected from wild birds in Asia and North America 2011–2015. A bootstrap analysis was performed with 500 replications; results are displayed at nodes. The analysis involved 78 nucleotide sequences. All positions containing gaps and missing data were eliminated. There was a total of 1679 positions in the final dataset. Evolutionary analyses were conducted in MEGA7. Sequences originating from Izembek National Wildlife Refuge, Alaska, USA, are identified by asterisks (***) following the strain name. Numerical clade names, provided next to brackets, represent the classification used in this study.
(EPS)

S15 Fig Phylogeny of HA H8 nucleotide sequences. Maximum likelihood phylogenetic tree based on the General Time Reversible model (G+I) depicting the inferred relationship of the complete coding region of HA H8 segment sequences from influenza A viruses collected from wild birds in Asia and North America 2011–2015. A bootstrap analysis was performed with 500 replications; results are displayed at nodes. The analysis involved 8 nucleotide sequences. All positions containing gaps and missing data were eliminated. There was a total of 1696 positions in the final dataset. Evolutionary analyses were conducted in MEGA7. Sequences originating from Izembek National Wildlife Refuge, Alaska, USA, are identified by asterisks (***) following the strain name. Numerical clade names, provided next to brackets, represent the classification used in this study.
(EPS)

S16 Fig Phylogeny of HA H9 nucleotide sequences. Maximum likelihood phylogenetic tree based on the General Time Reversible model (G+I) depicting the inferred relationship of the complete coding region of HA H9 segment sequences from influenza A viruses collected from wild birds in Asia and North America 2011–2015. A bootstrap analysis was performed with 500 replications; results are displayed at nodes. The analysis involved 54 nucleotide sequences. All positions containing gaps and missing data were eliminated. There was a total of 1679 positions in the final dataset. Evolutionary analyses were conducted in MEGA7. Sequences originating from Izembek National Wildlife Refuge, Alaska, USA, are identified by asterisks (***) following the strain name. Numerical clade names, provided next to brackets, represent the classification used in this study.
(EPS)

S17 Fig Phylogeny of HA H10 nucleotide sequences. Maximum likelihood phylogenetic tree based on the General Time Reversible model (G+I) depicting the inferred relationship of the complete coding region of HA H10 segment sequences from influenza A viruses collected from wild birds in Asia and North America 2011–2015. A bootstrap analysis was performed with 500 replications; results are displayed at nodes. The analysis involved 81 nucleotide sequences. All positions containing gaps and missing data were eliminated. There was a total of 1683 positions in the final dataset. Evolutionary analyses were conducted in MEGA7. Sequences originating from Izembek National Wildlife Refuge, Alaska, USA, are identified by asterisks (***) following the strain name. Numerical clade names, provided next to brackets, represent the classification used in this study.
(EPS)

S18 Fig Phylogeny of HA H11 nucleotide sequences. Maximum likelihood phylogenetic tree based on the General Time Reversible model (G+I) depicting the inferred relationship of the complete coding region of HA H11 segment sequences from influenza A viruses collected from wild birds in Asia and North America 2011–2015. A bootstrap analysis was performed with 500 replications; results are displayed at nodes. The analysis involved 75 nucleotide sequences. All positions containing gaps and missing data were eliminated. There was a total of 1691 positions in the final dataset. Evolutionary analyses were conducted in MEGA7. Sequences originating from Izembek National Wildlife Refuge, Alaska, USA, are identified by asterisks (***) following the strain name. Numerical clade names, provided next to brackets, represent the classification used in this study.
(EPS)

S19 Fig Phylogeny of HA H12 nucleotide sequences. Maximum likelihood phylogenetic tree based on the General Time Reversible model (G+I) depicting the inferred relationship of the complete coding region of HA H12 segment sequences from influenza A viruses collected from wild birds in Asia and North America 2011–2015. A bootstrap analysis was performed with 500 replications; results are displayed at nodes. The analysis involved 13 nucleotide sequences. All positions containing gaps and missing data were eliminated. There was a total of 1695 positions in the final dataset. Evolutionary analyses were conducted in MEGA7. Sequences originating from Izembek National Wildlife Refuge, Alaska, USA, are identified by asterisks (***) following the strain name. Numerical clade names, provided next to brackets, represent the classification used in this study.
(EPS)

S20 Fig Phylogeny of HA H13 nucleotide sequences. Maximum likelihood phylogenetic tree based on the General Time Reversible model (G+I) depicting the inferred relationship of the complete coding region of HA H13 segment sequences from influenza A viruses collected from wild birds in Asia and North America 2011–2015. A bootstrap analysis was performed with 500 replications; results are displayed at nodes. The analysis involved 31 nucleotide sequences. All positions containing gaps and missing data were eliminated. There was a total of 1693 positions in the final dataset. Evolutionary analyses were conducted in MEGA7. Sequences originating from Izembek National Wildlife Refuge, Alaska, USA, are identified by asterisks (***) following the strain name. Numerical clade names, provided next to brackets, represent the classification used in this study.
(EPS)

S21 Fig Phylogeny of HA H16 nucleotide sequences. Maximum likelihood phylogenetic tree based on the General Time Reversible model (G+I) depicting the inferred relationship of the complete coding region of HA H16 segment sequences from influenza A viruses collected from wild birds in Asia and North America 2011–2015. A bootstrap analysis was performed with 500 replications; results are displayed at nodes. The analysis involved 42 nucleotide sequences. All positions containing gaps and missing data were eliminated. There was a total of 1697 positions in the final dataset. Evolutionary analyses were conducted in MEGA7. Sequences originating from Izembek National Wildlife Refuge, Alaska, USA, are identified by asterisks (***) following the strain name. Numerical clade names, provided next to brackets, represent the classification used in this study.
(EPS)

S22 Fig Phylogeny of NA N1 nucleotide sequences. Maximum likelihood phylogenetic tree based on the General Time Reversible model (G+I) depicting the inferred relationship of the complete coding region of NA N1 segment sequences from influenza A viruses collected from wild birds in Asia and North America 2011–2015. A bootstrap analysis was performed with 500 replications; results are displayed at nodes. The analysis involved 184 nucleotide sequences. All positions containing gaps and missing data were eliminated. There was a total of 1344 positions in the final dataset. Evolutionary analyses were conducted in MEGA7. Sequences originating from Izembek National Wildlife Refuge, Alaska, USA, are identified by asterisks (***) following the strain name. Numerical clade names, provided next to brackets, represent the classification used in this study.
(EPS)

S23 Fig Phylogeny of NA N2 nucleotide sequences. Maximum likelihood phylogenetic tree based on the General Time Reversible model (G+I) depicting the inferred relationship of the complete coding region of NA N2 segment sequences from influenza A viruses collected from wild birds in Asia and North America 2011–2015. A bootstrap analysis was performed with 500 replications; results are displayed at nodes. The analysis involved 224 nucleotide sequences. All positions containing gaps and missing data were eliminated. There was a total of 1390 positions in the final dataset. Evolutionary analyses were conducted in MEGA7. Sequences originating from Izembek National Wildlife Refuge, Alaska, USA, are identified by asterisks (***) following the strain name. Numerical clade names, provided next to brackets, represent the classification used in this study.
(EPS)

S24 Fig Phylogeny of NA N3 nucleotide sequences. Maximum likelihood phylogenetic tree based on the General Time Reversible model (G+I) depicting the inferred relationship of the complete coding region of NA N3 segment sequences from influenza A viruses collected from wild birds in Asia and North America 2011–2015. A bootstrap analysis was performed with 500 replications; results are displayed at nodes. The analysis involved 156 nucleotide sequences. All positions containing gaps and missing data were eliminated. There was a total of 1404 positions in the final dataset. Evolutionary analyses were conducted in MEGA7. Sequences originating from Izembek National Wildlife Refuge, Alaska, USA, are identified by asterisks (***) following the strain name. Numerical clade names, provided next to brackets, represent the classification used in this study.
(EPS)

S25 Fig Phylogeny of NA N4 nucleotide sequences. Maximum likelihood phylogenetic tree based on the General Time Reversible model (G+I) depicting the inferred relationship of the complete coding region of NA N4 segment sequences from influenza A viruses collected from wild birds in Asia and North America 2011–2015. A bootstrap analysis was performed with 500 replications; results are displayed at nodes. The analysis involved 21 nucleotide sequences. All positions containing gaps and missing data were eliminated. There was a total of 1411 positions in the final dataset. Evolutionary analyses were conducted in MEGA7. Sequences originating from Izembek National Wildlife Refuge, Alaska, USA, are identified by asterisks (***) following the strain name. Numerical clade names, provided next to brackets, represent the classification used in this study.
(EPS)

S26 Fig Phylogeny of NA N5 nucleotide sequences. Maximum likelihood phylogenetic tree based on the General Time Reversible model (G+I) depicting the inferred relationship of the complete coding region of NA N5 segment sequences from influenza A viruses collected from wild birds in Asia and North America 2011–2015. A bootstrap analysis was performed with 500 replications; results are displayed at nodes. The analysis involved 40 nucleotide sequences. All positions containing gaps and missing data were eliminated. There was a total of 1418 positions in the final dataset. Evolutionary analyses were conducted in MEGA7. Sequences originating from Izembek National Wildlife Refuge, Alaska, USA, are identified by asterisks (***) following the strain name. Numerical clade names, provided next to brackets, represent the classification used in this study.
(EPS)

S27 Fig Phylogeny of NA N6 nucleotide sequences. Maximum likelihood phylogenetic tree based on the General Time Reversible model (G+I) depicting the inferred relationship of the complete coding region of NA N6 segment sequences from influenza A viruses collected from wild birds in Asia and North America 2011–2015. A bootstrap analysis was performed with 500 replications; results are displayed at nodes. The analysis involved 227 nucleotide sequences. All positions containing gaps and missing data were eliminated. There was a total of 1367 positions in the final dataset. Evolutionary analyses were conducted in MEGA7. Sequences originating from Izembek National Wildlife Refuge, Alaska, USA, are identified by asterisks (***) following the strain name. Numerical clade names, provided next to brackets, represent the classification used in this study.
(EPS)

S28 Fig Phylogeny of NA N7 nucleotide sequences. Maximum likelihood phylogenetic tree based on the General Time Reversible model (G+I) depicting the inferred relationship of the complete coding region of NA N7 segment sequences from influenza A viruses collected from wild birds in Asia and North America 2011–2015. A bootstrap analysis was performed with 500 replications; results are displayed at nodes. The analysis involved 64 nucleotide sequences. All positions containing gaps and missing data were eliminated. There was a total of 1403 positions in the final dataset. Evolutionary analyses were conducted in MEGA7. Sequences originating from Izembek National Wildlife Refuge, Alaska, USA, are identified by asterisks (***) following the strain name. Numerical clade names, provided next to brackets, represent the classification used in this study.
(EPS)

S29 Fig Phylogeny of NA N8 nucleotide sequences. Maximum likelihood phylogenetic tree based on the General Time Reversible model (G+I) depicting the inferred relationship of the complete coding region of NA N8 segment sequences from influenza A viruses collected from wild birds in Asia and North America 2011–2015. A bootstrap analysis was performed with 500 replications; results are displayed at nodes. The analysis involved 295 nucleotide sequences. All positions containing gaps and missing data were eliminated. There was a total of 1399 positions in the final dataset. Evolutionary analyses were conducted in MEGA7. Sequences originating from Izembek National Wildlife Refuge, Alaska, USA, are identified by asterisks (***) following the strain name. Numerical clade names, provided next to brackets, represent the classification used in this study.
(EPS)

S30 Fig Phylogeny of NA N9 nucleotide sequences. Maximum likelihood phylogenetic tree based on the General Time Reversible model (G+I) depicting the inferred relationship of the complete coding region of NA N9 segment sequences from influenza A viruses collected from wild birds in Asia and North America 2011–2015. A bootstrap analysis was performed with 500 replications; results are displayed at nodes. The analysis involved 68 nucleotide sequences. All positions containing gaps and missing data were eliminated. There was a total of 1389 positions in the final dataset. Evolutionary analyses were conducted in MEGA7. Sequences originating from Izembek National Wildlife Refuge, Alaska, USA, are identified by asterisks (***) following the strain name. Numerical clade names, provided next to brackets, represent the classification used in this study.
(EPS)
