# Supplementary material for: Influenza A virus recovery, diversity, and intercontinental exchange: A multi-year assessment of wild bird sampling at Izembek National Wildlife Refuge, Alaska
Source: PLoS One. 2018 Apr 5;13(4):e0195327. doi: 10.1371/journal.pone.0195327 (PMC5950690; doi:10.1371/journal.pone.0195327)
Supplement: S1 Table — Information below the diagonal line of shaded boxes in the table report p-values for comparisons of IAV sequence distributions across all clades (see Methods) within each phylogeny by gene segment. Above the diagonal line of shaded boxes are p-values for comparisons among host species groups with respect to the proportion of IAV sequences in Asian/mixed-origin clades (see Methods). P-values < 0.0083 and therefore interpreted as statistically significant are indicated in bold font. (DOCX) [file pone.0195327.s001.docx]

|  | Gene segment | northern pintail | other dabbling duck | emperor goose | glaucous-winged gull |
| --- | --- | --- | --- | --- | --- |
| northern pintail | all |  | **2.569E-04** | 4.763E-01 | **1.489E-04** |
| other dabbling duck | PB2 | 1.660E-02 |  | **3.250E-03** | **8.702E-10** |
|  | PB1 | 9.419E-01 |  |  |  |
|  | PA | 1.378E-02 |  |  |  |
|  | HA | **1.560E-03** |  |  |  |
|  | NP | 5.097E-01 |  |  |  |
|  | NA | 2.288E-02 |  |  |  |
|  | MA | 8.702E-01 |  |  |  |
|  | NS | 4.091E-01 |  |  |  |
| emperor goose | PB2 | 4.458E-02 | **8.200E-04** |  | 5.941E-02 |
|  | PB1 | 8.092E-01 | 5.371E-01 |  |  |
|  | PA | **7.840E-03** | 1.826E-01 |  |  |
|  | HA | 2.458E-01 | 3.202E-02 |  |  |
|  | NP | 2.892E-02 | 2.602E-02 |  |  |
|  | NA | 7.026E-01 | 5.491E-01 |  |  |
|  | MA | 1.000E+00 | 7.404E-01 |  |  |
|  | NS | 7.700E-01 | 4.702E-01 |  |  |
| glaucous-winged gull | PB2 | **2.800E-03** | **2.000E-05** | **1.640E-03** |  |
|  | PB1 | 3.978E-02 | 9.520E-03 | 2.353E-01 |  |
|  | PA | **4.720E-03** | **2.920E-03** | **8.000E-05** |  |
|  | HA | **2.000E-05** | **2.000E-05** | **3.400E-04** |  |
|  | NP | **2.000E-05** | **2.000E-05** | **2.600E-04** |  |
|  | NA | **2.000E-05** | **2.000E-05** | **2.400E-04** |  |
|  | MA | 1.950E-02 | 8.840E-02 | 1.082E-01 |  |
|  | NS | **2.000E-05** | **2.800E-04** | **4.000E-03** |  |
